#  The synergy between TB and HIV co-infection on perceived stigma in Ethiopia

**DOI:** 10.1186/1756-0500-3-249

**Published:** 2010-10-04

**Authors:** Amare Deribew, Yohannes HaileMichael, Markos Tesfaye, Dejene Desalegn, Ajeme Wogi, Shallo Daba

**Affiliations:** 1 Department of Epidemiology, Jimma University, Jimma, Ethiopia; 2 Department of Health Service management, Jimma University, Jimma, Ethiopia; 3 Department of psychiatry, Jimma University, Jimma, Ethiopia; 4 Nekemet Hospital, Nekemet, Ethiopia; 5 Oromiya Regional Health Bureau, Addis Ababa, Ethiopia

## Abstract

**Background:**

The synergy between tuberculosis (TB) and human immunodeficiency virus (HIV) co-infection on perceived stigma is not well studied. The objective of this study was to assess the effect of TB/HIV co-infection on perceived stigma in selected hospitals of Oromiya region, Ethiopia. A cross sectional study was conducted from February to April, 2009 in Adama, Nekemet and Jimma Specialized hospitals. Data were collected by trained HIV counselors. A structured questionnaire which consisted of socio-demographic variables, clinical information, perceived stigma, and depression was used to collect the data

**Findings:**

A total of 591 participants were included in the study of whom 124 (20.9%) were co-infected with TB/HIV. The stigma items were highly reliable (Cronbach's alpha = 0.93) and had strong inter dimension correlation. Respondents who were co-infected with TB and HIV were more likely to have perceived stigma compared to non-co-infected HIV patients, [OR = 1.4, (95% CI: 1.2, 2.0)]. Non-literate individuals [OR = 1.9, (95% CI: 1.2, 3.0)] and females [OR = 1.6, (95% CI: 1.2, 2.3)] had also more perceived stigma.

**Conclusions:**

TB/HIV co-infected patients, non-literate individuals and females were more likely to have high perceived stigma. Behavioral Change Communication should focus on these segments of the population to rectify the high perceived stigma.

## Background

The extensive nature of stigma against people with AIDS is well established [1,2]. People living with HIV/AIDS (PLHA) have been stigmatized worldwide since the beginning of the epidemic; leading to severe social consequences related to their rights, health care services, freedom, self identity and social interactions [1]. Stigma and discrimination related to HIV/AIDS would undermine public health efforts to combat the epidemic. The UN stated that stigma against PLHA still hamper prevention, care and treatment efforts everywhere [2].

The literature showed that perceived HIV stigma has a negative and constant impact on life satisfaction [3] and quality of life [4,5]. HIV related stigma has been correlated with poorer mental health outcomes, lowered self-esteem, lowered self-efficacy, and decreased adherence to antiretroviral treatments [6].

Several studies have been conducted on the consequences of stigma [7] and its effect on receiving HIV/AIDS related services [8,9]. However, little is known about the synergy between TB and HIV co-infection on perceived stigma. The present study assessed effect of TB/HIV co-infection on perceived stigma towards TB/HIV co-infection.

## Methods

From February to April, 2009, we conducted a cross sectional study in three hospitals in Ethiopia. A detail method of the study is published elsewhere [10]. In brief, the study was conducted in Adama, Nekemet and Jimma specialized hospitals located at the east, west and southwest part of Ethiopia. The study population consisted of 155 TB/HIV co infected and 465 HIV patients. During the study period, new TB patients (n = 124) in each TB/HIV clinic of the hospitals were included. For each TB/HIV co-infected patient, 3 HIV patients with no TB were included. The non-co-infected HIV patients were selected using a simple random sampling technique using the patients' unique identification number in the HIV clinics. The exclusion criteria for both groups included age less than 15 years and presence of known chronic illnesses like diabetes mellitus and hypertension.

A structured questionnaire was used to collect information from the respondents. The content of the questionnaire included socio-demographic variables, clinical information, social support, perceived stigma and depression. Perceived stigma was measured by 23 Likert scale items adopted from Berger et al [11].

Data were entered into computer and analyzed using SPSS version 15.0 software. The stigma questions were scaled in positive direction where higher score denotes high perceived stigma. A total stigma score was created by summing all the scores of the stigma questions. The mean was calculated from the total stigma score and individuals who scored below the mean were categorized as having 'low' perceived stigma. Those who scored above the mean were categorized as having 'high' perceived stigma. Bivariate analysis was computed to identify factors associated with perceived stigma. To control the effect of confounding variables, stepwise logistic regression model was done to identify predictors of perceived stigma. Variables which had statistically significant association in the bivariate analysis were candidates for the multivariate logistic regression model. Interaction between variables were tested but are not reported as none were significant.

Ethical clearance was obtained from Jimma University ethical review board. The purpose of the study was explained to the respondents. Written consent was obtained from the study participants. To ensure confidentiality, codes were anonymous.

## Results

Of the 620 eligible respondents, complete data were obtained from 591 (95%) of them. One hundred twenty four (20.9%) of the study participants were co-infected with TB and HIV. From the planned 155 TB/HIV co-infected patients, only 124 of them were interviewed and the rest 31 patients lost to follow up. Of the co-infected patients, 61 (49.2%) and 42 (33.8%) had smear negative and positive TB respectively. About two third (67.2%) of the patients had CD4 lymphocyte count of less than 200/μL. Of the total participants, 107 (18%) and 355 (60%) were in the 2^nd ^and 3^rd ^stage of the WHO clinical stage classification respectively.

The Majority, 344 (58.2%) of the study participants were females. The mean age of the respondents was 33.4 years (SD ± 8.1). Nearly half 276 (46.7%) of the respondents were in the age group of 25-34 years (Table-1).

**Table 1:** Socio-demographic characteristics of the study population in three hospitals of Oromia, March, 2009, (N = 591)

Variables	Number (%)
**Age in Years**	
15-24	54 (9.1)
25-34	276 (46.7)
> = 35	261 (44.2)
**Sex**	
Male	247 (41.8)
Female	344 (58.2)
**Educational status**	
Illiterate	104 (17.6)
literate	487 (82.4)
**Occupation**	
Government employee	68 (11.4)
Private employee	99 (16.8)
Merchant	79 (13.4)
Farmer	45 (7.6)
Housewives	94 (15.9)
Daily laborer	115 (19.5)
No Job	91 (15.4)

More than half of the respondents (56%) had high perceived stigma. The Multivariate analysis predicted that there was a significant relationship between perceived stigma and TB/HIV co-infection, depression, level of education and gender. Respondents who were co-infected with TB and HIV were 1.4 times more likely to have high perceived stigma as compared to non-co-infected HIV patients, [OR = 1.4, (95% CI: 1.2, 2.0)]. Non-literate individuals were 2 times more likely to experience high perceived stigma than literates,[OR = 1.9, (95% CI: 1.2, 3.0)]. Individuals who had depression were 2.3 times more likely to have high perceived stigma than non-depressed individuals [OR = 2.3;(95% CI: 1.5, 3.2)]. Women had higher perceived stigma than men [OR = 1.6, (95% CI: 1.2, 2.3)] (Table-2).

**Table 2:** Predictors of perceived stigma among HIV patients in three hospitals of Oromia, March, 2009

Variable	Perceived stigma		Adjusted OR(95%CI)
	High	Low	
**TB/HIV co-infection**			
Yes	70	54	1.4(1.1, 2.0)
NO	202	265	1
**Depression**			
Yes	165	132	2.3(1.5,3.2)
No	107	187	1
**Education**			
Illiterate	62	42	1.9(1.2, 3.0)
literate	210	277	
**Gender**			
Female	176	168	1.6(1.2, 2.3)
Male	96	151	1

Age, Social support, income, CD4 lymphocyte count, and WHO staging didn't have statistically significant association with perceived stigma.

## Discussion

This study identified factors associated with perceived stigma in HIV patients. More than half of our study participants had experienced high perceived stigma. This figure is much higher than the finding of Vissel et al (33%) in South Africa [12]. However, the burden of stigma by other people (negative reaction towards PLHA) might be lower than the perceived stigma in Ethiopia. A study done in India found that actual stigma was much lower than perceived stigma [13]. The high proportion of people with high perceived stigma in our setting could have negative consequences on adherence to antiretroviral therapy and other HIV/AIDS related care [8,9,13] and disclosure of the HIV status of individuals [14]. Self stigmatized people might develop negative attitude towards health professionals and health care and are less likely to disclose their HIV status and could spread the virus to others [14].

A study in South Africa showed that older individuals, males and the less educated ones were more likely to had high perceived stigma [12]. In our study, non-literate individuals and females were more likely to have high perceived stigma. The low social status and economic dependency of women and non-literate individuals in our country could act in synergy with TB/HIV co-infection to increase the perceived stigma among women and non-literate individuals. Unlike other studies [14,15], we did not show an association between age and income with perceived stigma. The literature showed that social support is a good buffer in reducing perceived stigma [15]. However, our study couldn't prove this hypothesis.

People with mental health problems are at higher risk of experiencing perceived stigma [15,16]. However, in our study, stigma could be also the risk factor for depression. Internalized shame and feeling of guilty is one of the predictor of depression or other form of mental distress [16].

TB/HIV co-infected patients were more likely to have perceived stigma. The literature showed that people with TB have been discriminated and isolated from the society for fear of infection [17]. Co-infected patients might perceive that the community would avoid them for fear of infection in our setting. This double burden stigma of TB might aggravate the already existing perceived stigma due to HIV. WHO staging and lower CD4 lymphocyte count didn't show significant association with perceived stigma in our setting. Other literature showed that clinical stage was one of the predictors of perceived stigma [15].

Although we tried to document the effect of TB/HIV co-infection on perceived stigma for the first time, our finding didn't include survey on actual stigma and qualitative study to triangulate the findings.

## Conclusions

More than half of the study participants had high perceived stigma. Non-literate individuals, females and TB/HIV coinfected patients were more likely to have high perceived stigma. Behavioral Change Communication should focus on these segments of the population to reduce perceived stigma.

## Competing interests

The authors declare that they have no competing interests.

## Authors' contributions

**AD **Conceived the study and was involved in the design, analysis and report writing. **YH **was involved in data analysis and report writing. **MT **was involved in the design of the study and critically reviewed the article. **AW**, **SD **and **DD **were involved in field activities and reviewed the article. All authors read and approved the manuscript.

## References

[B1] Mawar N Sahay S Pandit A Mahajan U The Third phase HIV pandemic: Social consequences of HIV/AIDS stigma & discrimination & future needs Indian J Med Research 2005 122 471 484 16517997

[B2] WHO/UNAIDS Progress on Global Access to HIV Antiretroviral therapy: A report on "3 by 5" and beyond 2006 WHO, Geneva

[B3] Greeff M Leana R Wantland D Makoae L Chirwa M Dlamini P Kohi T Mullan J Perceived HIV stigma and life satisfaction among persons living with HIV infection in five African countries: A longitudinal study Int J Nurs Stud 2009 in press 19854440 10.1016/j.ijnurstu.2009.09.008 PMC2832093

[B4] Buseh AG Kelber ST Hewitt JB Stevens PE Park CG Perceived stigma and life satisfaction: Experiences of urban African American men living with HIV/AIDS International Journal of Men's Health 2006 5 1 35 51 10.3149/jmh.0501.35

[B5] Perlick DA Rosenheck RA Clarkin JF Sirey JA Salahi J Struening EL Link BG Stigma as a barrier to recovery: adverse effects of perceived stigma on social adaptation of persons diagnosed with bipolar affective disorder Psychiatr Serv 2001 52 1627 1632 10.1176/appi.ps.52.12.1627 11726754

[B6] Baird ST Bogart LM Delahanty DL Health-related correlates of perceived discrimination in HIV care AIDS Patient Care and STDs 2004 18 1 19 26 10.1089/108729104322740884 15006191

[B7] Chesney MA Smith AW Critical delays in HIV testing and care American Behavioral Scientist 1999 42 1158 1170 10.1177/00027649921954822

[B8] WHO Treat 3 million by 2005-a public health approach for scaling up antiretroviral (ARV) treatment: a toolkit for programme managers 2003 WHO, Geneva 1 91

[B9] Lekas H Siegel K Schrimshaw E Continuities and Discontinuities in the Experiences of Felt and Enacted Stigma among Women with HIV/AIDS Qual Health Res 2006 16 1165 90 10.1177/1049732306292284 17038751

[B10] Deribew A Tesfaye M Hailemichael Y Negussu N Apers L Colebunders R TB/HIV co-infection: its impact on Quality of life Quality of Life and Health Outcomes 2009 in press 10.1186/1477-7525-7-105 PMC2809048 20040090

[B11] Berger B Ferrans C Lashley F Measuring Stigma in People with HIV: Psychometric Assessment of the HIV Stigma Scale Research in Nursing & Health 2001 24 518 529 10.1002/nur.10011 11746080

[B12] Vissel MJ Makin JD Vandormael A Sikkema KJ Forsyth BW HIV/AIDS stigma in a South African community AIDS Care 2009 21 2 197 206 10.1080/09540120801932157 19229689 PMC4238924

[B13] Thomas BE Rehman F Suryanarayanan D Josephine K Dilip K DOrairaj VS Swaminathan S How stigmatizing is stigma in the life of people living with HIV: A study on HIV positive individuals from Chennai, South India AIDS Care 2005 17 7 795 801 16120496 10.1080/09540120500099936

[B14] Smith R Rossetto K Peterson B A meta-analysis of disclosure of one's HIV-positive status, stigma and social support AIDS Care 2008 20 10 1266 1275 10.1080/09540120801926977 18608080

[B15] Logie C Gadalla TM Meta analysis of health and demographic correlates of stigma towards people living with HIV AIDS Care 2009 21 6 742 53 10.1080/09540120802511877 19806490

[B16] Li Li Lee S Thammawijaya P Jiraphongsa C Rotheramborus M Stigma, social support, and depression among people living with HIV in Thailand AIDS Care 2009 21 8 1007 13 10.1080/09540120802614358 20024757 PMC2803757

[B17] Dodor EA Kelly S 'We are afraid of them': Attitudes and behaviors of community members towards tuberculosis in Ghana and implications for TB control efforts Psychology, Health & Medicine 2009 14 2 170 179 10.1080/13548500802199753 19235076

